# Surveillance of Salmonella and antimicrobial resistance in industrial poultry enterprises: biofilm-forming strains and critical control points

**DOI:** 10.1099/jmm.0.001993

**Published:** 2025-03-25

**Authors:** Birzhan Biyashev, Aygerim Zhusanbayeva, Zhumagul Kirkimbayeva, Asel Zholdasbekova, Dinara Sarybayeva

**Affiliations:** 1 Department of Microbiology, Virology and Immunology, Kazakh National Agrarian Research University, Almaty, Republic of Kazakhstan

**Keywords:** food safety, infection, pathogenicity, poultry farm, systematic observation

## Abstract

**Introduction.***Salmonella* contamination in the poultry industry poses substantial health risks, especially due to biofilm-forming strains that resist disinfection and antibiotic treatment. Biofilm-forming *Salmonella* strains are particularly challenging to control, as they adhere to surfaces in production environments, leading to persistent contamination. This study assesses the prevalence of *Salmonella*, examines antibiotic resistance patterns and evaluates biosecurity effectiveness at poultry farms in Kazakhstan.

**Hypothesis/Gap Statement.** There is limited data on the prevalence and antibiotic resistance of biofilm-forming *Salmonella* strains in Kazakhstan’s poultry industry, highlighting a need to characterize these strains to inform effective control measures.

**Aim.** The purpose of this study was to systematically identify and characterize *Salmonella* strains, including biofilm-forming types, within industrial poultry enterprises in Kazakhstan.

**Methodology.** A total of 660 samples were collected from various poultry production sites, including feed, water sources, cloacal flushes and shoe covers. *Salmonella* detection followed standardized protocols, and antibiotic sensitivity of identified strains was analysed to evaluate resistance patterns.

**Results.***Salmonella* was detected in 11.5% (95% CI) of the 660 samples, with the highest contamination observed in shoe covers, cloacal flushes, feed and water. This prevalence rate indicates a significant presence of the pathogen in the country’s poultry production chain, falling between the higher rates seen in countries like China (22.2%) and Egypt (29.1%) and the lower rates observed in countries like Brazil (3.4%). The most prevalent strain was *Salmonella gallinarum-pullorum* (61.8%), followed by *Salmonella typhimurium* (18.4%) and *Salmonella enteritidis* (14.5%). Antibiotic sensitivity analysis revealed that *S. gallinarum-pullorum* was largely susceptible to common antibiotics, while *S. typhimurium* displayed considerable resistance, emphasizing the need for alternative treatments.

**Conclusion.** The findings underscore the importance of strict sanitary and hygiene standards throughout poultry production, with a particular focus on managing biofilm-forming *Salmonella* strains. Implementing comprehensive Hazard Analysis and Critical Control Points protocols is essential to address contamination hotspots effectively. Future studies should investigate genetic mechanisms underlying biofilm formation and resistance in *Salmonella* strains to inform targeted interventions, ultimately improving food safety and public health outcomes.

­

**Impact Statement**

This study underscores the critical need for stringent biosecurity measures at poultry enterprises in Kazakhstan by revealing significant *Salmonella* contamination, including biofilm-forming strains that resist standard disinfection methods. The study highlights vulnerabilities in current infection control practices and suggests urgent updates to sanitation protocols. The findings provide essential insights for improving food safety in Kazakhstan’s poultry industry and minimizing health risks associated with antibiotic-resistant pathogens.

## Data Availability

The data that support the findings of this study are available on request from the corresponding author.

## Introduction

*Salmonella* is a significant global health burden due to its ability to cause severe foodborne infections, often through contaminated poultry products. Transmission commonly occurs through the consumption of contaminated meat or eggs, allowing the bacteria to enter the gastrointestinal tract, where they can cause symptoms ranging from mild gastroenteritis to severe systemic infections. *Salmonella* infections manifest primarily as abdominal pain, diarrhoea, nausea and vomiting. Vulnerable populations, including young children, the elderly and immunocompromised individuals, face heightened risks, making the control of *Salmonella* contamination in food sources an urgent public health priority.

*Salmonella* is a major global issue in poultry farming, as it frequently contaminates meat and eggs, leading to foodborne illnesses. The prevalence of *Salmonella* in poultry varies widely: in regions with strict biosecurity measures, such as parts of Europe and North America, it tends to be lower (e.g. 8.3% in Spain, 3.4% in Brazil), while in areas with less regulatory oversight, like China and Egypt, it reaches as high as 22.2 and 29.1%, respectively [1]. Antibiotic overuse in many countries has contributed to the emergence of resistant *Salmonella* strains, which complicates treatment and heightens public health risks. Additionally, *Salmonella*’s ability to form biofilms on surfaces in poultry facilities allows it to persist despite cleaning efforts, contributing to ongoing contamination challenges. Addressing *Salmonella* in poultry farms requires enhanced biosecurity practices, regulatory enforcement, antimicrobial control and research into alternative measures such as biofilm prevention, vaccines and probiotics to improve food safety and reduce infection risks.

A growing concern in the battle against *Salmonella* is the bacteria’s evolving antimicrobial resistance. The use of antibiotics in poultry farming, often as a preventative measure or growth promoter, has particularly accelerated this issue. Many *Salmonella* serovars now display resistance to critical antimicrobials, including fluoroquinolones and cephalosporins, which are essential for treating severe infections. The serovar (serotype) is a distinct strain within a micro-organism species, identified by unique surface antigens that trigger specific immune responses. The genus *Salmonella* includes more than 2,600 known serovars. Of particular concern are strains that produce extended-spectrum beta-lactamases, rendering them resistant to beta-lactam antibiotics. This resistance complicates treatment options, especially in countries where the regulatory framework for antimicrobial use is still developing [1].

In Kazakhstan, the poultry industry plays an important role in meeting the country’s meat consumption demands, as chicken remains the most accessible and affordable source of protein. According to data from the Bureau of National Statistics of the Agency of Strategic Planning and Reforms of the Republic of Kazakhstan [2], there are ~48,424 thousand poultry units in the country, with Northern Kazakhstan accounting for a significant share of production. Research indicates that *Salmonella* enterica serovars, particularly *Salmonella gallinarum-pullorum*, *Salmonella typhimurium* and *Salmonella enteritidis*, are among the main causative agents of foodborne disease outbreaks worldwide [3,5]. Biofilm-forming strains of *Salmonella*, which are highly resilient to standard disinfection and antibiotic treatments, are particularly problematic in Kazakhstan’s poultry sector, where inadequate regulation of antimicrobial use exacerbates the risk of widespread contamination [6]. These biofilm-forming strains not only persist on production surfaces but also demonstrate a significant capacity to evade conventional sanitation protocols.

Studies by Mendybayeva *et al*. [7] and Barmak *et al*. [8] underscore the importance of biofilm in protecting *Salmonella* from environmental stressors, facilitating survival and promoting antimicrobial resistance. Yet, much remains unclear regarding the specific environmental conditions and surface types that facilitate biofilm formation in poultry farms. Furthermore, existing studies have often focused on isolated serotypes or specific antimicrobial agents, limiting our understanding of multidrug-resistant strains’ full spectrum in Kazakhstan [9,10]. This knowledge gap underscores the need for systematic studies to identify contamination hotspots, characterize *Salmonella* species and their resistance profiles and evaluate current biosecurity practices.

Given these gaps, this study hypothesizes that *Salmonella* contamination at poultry farms in Kazakhstan is both widespread and facilitated by the prevalence of biofilm-forming, antibiotic-resistant strains. Therefore, the objective of this study was to conduct a comprehensive analysis of *Salmonella* distribution, serotype prevalence and antimicrobial resistance in Kazakhstan’s poultry farms. Specifically, this study aims to identify the most contaminated sites within the poultry production chain, characterize the morphological and resistance traits of detected *Salmonella* strains and assess the effectiveness of current control measures.

## Methods

### Study design and sample collection

From 2022 to 2024, 660 samples were collected from multiple points within poultry production facilities across Kazakhstan to detect *Salmonella*. Specifically, 180 samples were taken from cloacal flushes, 160 from shoe covers, 140 from droppings and 100 from drinking water, which included both reservoir and nipple system sources. Additionally, 80 samples were collected from equipment, transport vehicles and egg containers, while the remaining 100 samples were obtained from finished poultry products, including packaged meat and organs. Sampling focused on high-risk areas: cloacal flushes, shoe covers, droppings and drinking water (from both reservoirs and nipple systems). Flushes were also taken from equipment, transport vehicles and egg containers, as these surfaces frequently contact potential contaminants. Finished products, including packaged meat and organs, were sampled to assess end-product contamination risks. All samples were stored at 4 °C immediately after collection to inhibit bacterial growth until lab processing. Isolation followed standardized protocols to ensure reliable *Salmonella* detection. This systematic approach enabled an accurate assessment of contamination hotspots across production stages, informing targeted control and biosecurity measures.

### *Salmonella* detection and identification

*Salmonella* detection and identification followed Interstate standards GOST R 53665-2009 [11] and GOST 31659-2012 [12] for poultry meat, edible offal and poultry meat ready-to-cook products. The non-selective enrichment stage used standard buffered peptone water as a storage nutrient medium. Biomaterial samples were combined with the medium in a 1:10 ratio and incubated at 37±1 °C for 18±2 h. For selective enrichment, 1 cm^3^ of the enriched medium was transferred to 9 cm^3^ of tetrationate broth and incubated at 37±1 °C for 24±3 h. Following enrichment, cultures were transferred to various agarized nutrient media, including *Salmonella*-Shigella (SS) agar, xylose-lysine-deoxycholate (XLD) agar, Endo, Levin, Ploskirev and bismuth sulphite agar. These plates were incubated at 37±1 °C for 24–48 h. Characteristic colonies were sampled, Gram-stained according to Interstate standard GOST 21237-75 [13] and microscopically examined.

For the biochemical identification process, colonies were suspended in saline solution to achieve a turbidity matching 1 McFarland unit. The suspension was then analysed for various biochemical properties, including enzymatic activity, hydrogen sulphide production, indole formation and the ability to metabolize citrate and aesculin. Haemolytic characteristics were evaluated by culturing the samples on blood agar containing 7% sheep blood. The resulting patterns were classified as *α*-haemolysis (transparent zones), *β*-haemolysis (greenish zones) or *γ*-haemolysis (no zones). Serogroup identification of *Salmonella* was performed using pure cultures grown on slanted meat-peptone agar (MPA). The serological testing employed slide agglutination reactions using a comprehensive set of sera, including *Salmonella* O-complex and monoreceptor O- and H-agglutinating sera, enabling precise serogroup determination. In cases where agglutination was absent despite other typical *Salmonella* characteristics, virulence was assessed through bioassays using white laboratory mice. These bioassays also served to compare the virulence of *Salmonella* strains isolated from different sources. All animal experiments were conducted in compliance with the European Convention for the Protection of Vertebrate Animals Used for Experimental and Other Scientific Purposes [14] and the Universal Declaration on Animal Welfare [15].

### Antibiotic sensitivity testing

The sensitivity of micro-organisms to antibiotics was assessed using the disco diffusion method on Muller–Hinton agar (HiMedia, India), using standard paper discs (BD DDL, USA). The test procedure corresponded to the methodological guidelines MG 4.2.1890-04 [16]. For this purpose, a standard suspension was prepared from a daily culture of micro-organisms at a concentration of 1.510.8 c.f.u. cm^−3^, which corresponds to 0.5 McFarland. The cultures were kept in an incubator at a temperature of 35 °C for 20–24 h. The assessment of antibiotic sensitivity was carried out in accordance with the current guidelines on antibiotic sensitivity.

### Biofilm formation assessment

The ability of biofilm formation on plastic surfaces for each isolate was studied using 24-well polystyrene microtitre plates. The study was conducted according to adapted protocols, which allowed evaluating the ability of micro-organisms to adhere and form structured communities on synthetic material [17]. An experiment to assess the ability of isolates to form biofilms was conducted three times on different days for each sample. The final biofilm formation capacity of each isolate was determined by subtracting the OD of the negative control from the OD values of each isolate, where the final OD500 value was calculated as the difference between the average OD value of the isolate and the average OD value of the negative control. Data on the ability to form biofilms were additionally analysed by categorizing them based on final OD values: isolates with a final OD600 value less than 0.1 were classified as weak producers, those with an OD500 value in the range from 0.2 to 0.5 were recognized as moderate producers and isolates with an OD600 value above 0.5 were considered strong biofilm producers.

## Results

In total, from 2022 to 2024, 660 samples were collected from various enterprises of the industrial poultry industry of the republic, including biomaterial from sick and dead birds from poultry farms and private farmsteads. The samples were stored at 4 °C and delivered to the laboratory for subsequent isolation. The samples for the study included cloacal flushes, shoe covers from poultry houses, droppings, samples of drinking water from a reservoir, water from a nipple, flushes from equipment, flushes from transport for transporting chickens, transport underpads for day-old chickens, flushes from transport for transporting eggs and containers for transporting incubation eggs, incubation eggs, samples of finished products in packaging (meat, liver, heart), day-old chickens and pathological material.

The largest number of samples studied was noted for incubation eggs. In other cases, the number of samples varied, but in most of them, it was significantly less. It is noteworthy that the number of samples with detected *Salmonella* in all types of samples was significantly lower compared to the total number of samples. Cloacal chambers also accounted for the largest number of cases of *Salmonella* detection. A small number of tested samples and minimal cases of *Salmonella* detection were noted in chickens and in transport. An increase in the number of samples examined was observed in transport cells and cloacal flushes, but cases of *Salmonella* detection remained rare. Dust and fluff showed a significant increase in the number of samples examined and a moderate number of cases of *Salmonella* detection. In other types of samples, the number of samples examined varied, but the cases of *Salmonella* detection were minimal.

Thus, it was clearly demonstrated that the greatest risk of *Salmonella* detection was associated with cloacal chambers, which required additional attention and preventive measures. The remaining types of samples showed lower values both in terms of the number of samples examined and the number of cases of *Salmonella* detection but also required regular systematic observation and control to prevent the spread of infections. Next, it was important to consider the ratios between the samples according to the degree of detection of bacteria. This allowed the study to determine which types of samples were most susceptible to *Salmonella* infection. The analysis of such ratios revealed potential sources of infection and helped in analysing the degree of development of their population. Understanding these ratios was critical for an in-depth study of the epidemiological situation and the development of scientific recommendations (Table 1). When it turned out that cloacal chambers were significantly more likely to contain *Salmonella*, this indicated the need for a detailed study of sanitary conditions in incubators. Such information was extremely important for optimizing disinfection techniques and understanding the ways of infection spread.

**Table 1.** Distribution of samples according to the degree of detection of *Salmonella* bacteria

Sample type	Detection rate of *Salmonella* (%)
Incubation eggs	10
Transport underpad	5
Cloacal flushes	30
Dust, fluff	8
Shoe cover samples	18
Meconium	3
Equipment flushes	3
Egg warehouse	1
Water from the nipple	8
Feed	3
Finished goods	4
Pathological material from dead birds	7

In addition, data on the distribution of bacteria across different types of samples helped in the development of more effective research strategies. This allowed focusing resources and efforts on the most problematic areas, which made the learning process more focused and effective. Thus, the analysis of the ratios between samples not only improved understanding of the epidemiological situation but also contributed to deepening knowledge about the spread and development of *Salmonella* populations, helping to prevent further spread of infection.

Of the total number of materials studied, *Salmonella* bacteria were detected in 11.5% of the samples, which indicates the significant presence of this pathogen in the samples under study. The data obtained help to identify the most common sources of *Salmonella* contamination at all stages of the technological process of production and transportation of products. In poultry enterprises, the spread of *Salmonella* does not exclude the possibility of vertical transmission of the pathogen through an incubation egg, which is confirmed by the detection of the pathogen in 3.2% of incubation egg samples. Further studies also showed the presence of *Salmonella* in adult birds, which is confirmed by positive results of analysis of cloacal samples in 30% of cases. This indicates that an incubation egg can be a significant factor in the spread of the pathogen, which leads to infection of adult birds and, accordingly, to further contamination of products.

Possible sources of secondary contamination of products are production equipment and tables for processing carcasses, meat and offal. Studies show that *Salmonella* was detected in 4.25% of samples from equipment and work surfaces. This underlines the need for strict compliance with sanitary and hygienic standards at all stages of the technological process, since insufficient attention to the cleanliness of equipment can lead to a significant increase in the risk of contamination of products. As a result of the analyses, cases of the presence of *Salmonella* in the final product were identified, and this figure is 16.7%. These results emphasize the need for regular systematic observation and control of the sanitary condition of both production facilities and the equipment itself and compliance with strict sanitary standards during transportation of products.

According to the presented data, the greatest probability of detecting *Salmonella* is from samples taken from the body of birds, namely from cloacal flushes. This indicates that the main source of *Salmonella* contamination of products is the birds themselves. Consequently, measures to control poultry health and prevent infections play a key role in reducing contamination and ensuring the safety of end products. Thus, the results of this study emphasize the need for an integrated approach to controlling the spread of *Salmonella* in poultry enterprises, including measures to prevent vertical transmission of the pathogen, regular systematic observation of the sanitary condition of equipment and work surfaces and careful observation of bird health. Only by observing all these measures can the risk of *Salmonella* contamination of products be significantly reduced and food safety for consumers be ensured.

According to the presented data, various strains of *Salmonella* were isolated in poultry farms of the Republic of Kazakhstan, among which the most common was *S*. *gallinarum-pullorum*, which makes up 61.8% of the total number of isolates (Table 2). This type of *Salmonella* is a significant pathogen for birds, causing serious diseases and losses in poultry farms. Special attention should be paid to measures to control and prevent the spread of this pathogen.

**Table 2.** Results of typing of *Salmonella* isolates

No. of samples (*n*)	No. of positive samples, *n* (%)	Of these, *n* (%)			
		*S*. *typhimurium*	*S*. *enteritidis*	*S*. *gallinarum-pullorum*	*S. enterica*
660	76 (11.5)	14 (18.4)	11 (14.5)	47 (61.8)	4 (5.3)

*S. typhimurium* strains accounted for 18.4% of the total number of isolated cultures. This type of *Salmonella* is widespread and can infect both birds and other animals, including humans. It is important to note that most cultures of *S. typhimurium* were isolated from feed and shoe samples, which emphasizes the need for strict control over feed quality and compliance with sanitary and hygienic standards when working with poultry. *S. enteritidis*, another significant pathogen, accounted for 11.5% of the total number of *Salmonella* isolates. This type of *Salmonella* also poses a threat to the health of both birds and humans, causing diseases associated with the use of contaminated products.

It should be noted that 5.3% of the cultures had morphological, cultural and biochemical properties typical of *Salmonella*, but they were not agglutinable with available serums. This indicates the possible presence of new or atypical strains of *Salmonella*, which requires additional research to identify them and develop appropriate control methods. During the pathoanatomical examination of 26 fallen birds, in 8 cases, a pattern characteristic of salmonellosis was noted – an increase and blood filling of the liver and spleen, the presence of numerous spot haemorrhages in them, sometimes necrotic foci in some birds and an inflammatory process in the lungs and intestines were noted. Fibrinous ulcerative lesions of the mucous membrane of certain parts of the intestine were found in two birds. The gallbladder is enlarged and contains thick dark green bile. Such corpses were emaciated; muscle atrophy was observed. *Salmonella* was isolated from parenchymal organs and the gastrointestinal tract during bacteriological examination. A total of 47 *Salmonella* were isolated and identified during the study period. These strains demonstrated typical morphological, cultural-biochemical and serological properties, which allowed them to be uniquely identified. Most of the isolated strains belonged to two serological groups: B and D.

Morphologically isolated strains were small ovoid Gram-negative bacteria. When grown on various nutrient media, *Salmonella* strains showed characteristic cultural features. On meat-peptone broth, they caused the formation of a slight turbidity, which indicates their activity and reproduction in the medium. However, turbidity was more pronounced in the medium of tryptic soy broth (TSB), which indicates a more intensive growth of bacteria in this nutrient medium. On MPA, *Salmonella* strains grew to form small dewy colonies with a bluish tinge, which is a characteristic feature for the identification of these bacteria. This phenomenon is the result of the reaction of *Salmonella* to the components of the agar medium and is used to differentiate them from other micro-organisms.

On SS agar, *Salmonella* grew, forming round, colourless colonies with a dark top, which is characteristic of these bacteria and helps in their differentiation from other intestinal pathogens. These colonies stand out for their unique appearance, which facilitates the identification of *Salmonella* when using this nutrient medium. On the XLD agar medium, *Salmonella* colonies also had specific features: they were small, transparent and round with a black centre. This characteristic staining is conditioned by the ability of *Salmonella* to decarboxylate lysine and reduce sulphur, which leads to the formation of a black precipitate of iron sulphide in the centre of the colony. Due to the fact that haemolytic and DNase activity are among the main factors of *Salmonella* pathogenicity, these properties were investigated in the obtained isolates. The haemolytic activity of *Salmonella* is associated with their ability to destroy red blood cells, which promotes the spread of bacteria in the body and increases their pathogenicity. DNase activity, in turn, is conditioned by the ability of bacteria to decompose DNA, which can disrupt the normal functioning of host cells and contribute to the development of the infectious process.

Experiments on blood agar were conducted to study the haemolytic activity of isolated *Salmonella* isolates. The results showed that a significant part of the strains has pronounced haemolytic activity, which is confirmed by the presence of haemolysis zones around the colonies. This indicates the high pathogenic potential of these isolates and their ability to cause serious infectious processes. A DNA agar medium was used to study the DNase activity. The isolated *Salmonella* isolates showed varying degrees of activity in DNA decomposition, which was confirmed by the formation of transparent zones around colonies on DNA agar. The study of haemolytic and DNase activity among *Salmonella* isolates revealed that out of a total of 47 isolates, 16 (34%) exhibited haemolytic activity. Additionally, DNase activity was detected in 11 isolates, accounting for 23.4% of the total samples. These findings indicate that a notable proportion of the isolates possess virulence factors associated with tissue degradation and pathogenicity.

Haemolysis was formed on blood agar by 34% of the isolated strains; the production of the DNase enzyme was noted in 23.4% of cultures. When analysing the results obtained, it was found that it isolated from pathological material had haemolytic and DNase activity, respectively, 69 and 34%. To investigate the virulence of *Salmonella* isolates, 24 cultures from different isolation sources were selected. In experiments on white mice (*n*=5), the virulence of *Salmonella* isolates was assessed based on LD₁₀₀ values. Out of a total of 16 isolates, 2 exhibited virulence at a dose of 10³ c.f.u., while 4 isolates showed virulence at 10⁵ c.f.u. Another four isolates demonstrated lethal effects at 10⁷ c.f.u., and the remaining six isolates were found to be virulent at the highest dose of 10⁹ c.f.u.

Notably, virulence did not depend on the serovar or the serological group to which the studied strain of *Salmonella* belongs. The infecting dose of *Salmonella*, at which all experimental animals died, was higher in strains isolated from the clinical form of salmonellosis, whereas cultures isolated from environmental objects, water and cloacal flushes were lower (107–109 c.f.u. ml^−1^). Probably, the decrease or absence of virulence in strains isolated from the environment is associated with the adaptation of pathogens to living conditions outside the body. Once in the host’s body, they can restore their pathogenic properties and cause clinical forms of the disease. At the same time, virulence was also studied in those strains that could not be typed using the serological method. Non-agglutinable two strains were found to cause the same pathological and anatomical changes in mice as when they were infected with typical *Salmonella* strains.

Next, biofilm-forming strains among *Salmonella* isolates were identified, and the level of antibiotic resistance among them was determined. It is known that biofilm formation in bacteria is directly related to their ability to adhere to various surfaces. In this regard, studies were carried out to determine the adhesive properties of *Salmonella* to the surface of polystyrene plates. These studies were carried out in three repetitions. Suspensions of *Salmonella* cultures were previously prepared at TSB (Merck^®^). The concentration was brought to ~107 c.f.u. in a volume of 200 µl. Then the bacterial suspension was introduced into a 96-polystyrene plate and incubated for 2 h at 37 °C. To remove persistent bacteria, the plates were washed three times at 100 r.p.m. The adhered *Salmonella* was collected by scraping off the wells of the plates. A sample of the resulting cell suspension was diluted in 100 ml of saline solution and seeded onto the surface of Tryptic Soy Agar (TSA). After 20–24 h of incubation, colonies were counted in the thermostat (Table 3).

**Table 3.** Comparative data of different *Salmonella* serotypes on the ability to adhere and biofilm formation

Indicator	*S*. *typhimurium*	*S*. *enteritidis*	*S*. *gallinarum-pullorum*
Number of strains	14	11	47
Including (*n*/%)			
Strains – weak biofilm producers*	7/50	4/36.4	10/21.3
Strains – moderate biofilm producers**	4/28.57	4/36.4	14/29.8
Strains – strong biofilm producers***	3/21.42	3/27.2	23/48.9
Concentration of the original culture	10^7^	10^7^	10^7^
Number of adhered *Salmonella*	1.4×10^3^±235	0.9×10^3^±235	9.7×10^3^±235

Note: when measuring coloured wells on a spectrophotometer, OD is given: * – with a value of OD600<0.3; ** – with an OD600 value from 0.3 to 0.6; *** – with an OD600 value >0.6.

According to the results of testing of isolated *Salmonella*, *S*. *gallinarum-pullorum* has the greatest ability to adhere and form biofilm. In accordance with the data obtained and considering the fact that the maximum number of isolates isolated from various facilities in poultry farms in the country correspond to the *S. gallinarum-pullorum* serotype, it can be concluded that there is a correlation between these indicators, and it can be assumed that biofilm production may be a potential means of survival of * S*. *gallinarum-pullorum* in industrial poultry farming. The sensitivity of *Salmonella* strains to antimicrobial drugs was determined by the disco-diffuse method using HiDiscs cartridges with antibiotics manufactured by HiMedia Laboratories.

The presented results showed that *Salmonella* of various serovars are sensitive to the action of antibiotics, which is important for the development of effective strategies for the treatment and prevention of infections. Among the dominant serovars, the largest number of sensitive strains was identified among *S. gallinarum-pullorum*. This species has demonstrated significant susceptibility to most of the antibiotics used, which makes it possible to successfully use standard antimicrobials to treat infections caused by this serovar. Unlike *S. gallinarum-pullorum*, *S*. *typhimurium* strains have shown a higher degree of antibiotic resistance. The number of positive results confirming antibiotic resistance was significantly higher when testing strains of this particular serovar. Such resistance makes it difficult to treat infections caused by *S*. *typhimurium* and requires alternative therapeutic approaches or the use of more powerful and possibly more toxic antibiotics.

These results highlight the need for regular systematic observation of the antibiotic sensitivity of various *Salmonella* serovars to timely identify changes in their resistance and adjust treatment regimens. This is especially important in conditions of intensive poultry farming, where the spread of resistant strains can lead to serious economic losses and a threat to animal and human health.

Regular monitoring and testing of poultry flocks for *Salmonella* is essential in managing the risk of outbreaks, as early detection allows for immediate intervention to contain potential contamination. Consistent surveillance helps to identify sources of infection, track the presence of biofilm-forming strains and assess antimicrobial resistance patterns. By implementing a robust monitoring protocol, poultry farms can promptly respond to any increase in contamination, ultimately safeguarding animal and human health.

In addition to routine testing, adopting a Hazard Analysis and Critical Control Points (HACCP) system across all production stages is highly effective in controlling *Salmonella* contamination. The HACCP approach systematically identifies and addresses critical points where contamination may occur, enabling targeted and timely corrective actions. Integrating HACCP with biosecurity protocols reinforces the control over contamination sources, such as feed, water and equipment, thereby significantly reducing the risk of *Salmonella* spread. These proactive measures not only enhance food safety standards but also contribute to the overall quality and marketability of poultry products.

## Discussion

The prevalence of *Salmonella* in poultry products in Kazakhstan, observed at 11.5%, highlights a significant public health concern. This prevalence rate is lower than in countries like China, where *Salmonella* prevalence reaches 22.2% according to Karabasanavar *et al*. [18], and Egypt, where it is even higher at 29.1% as noted by Ashrafudoulla *et al*. [19]. However, it exceeds rates observed in Brazil, where Siddique *et al*. [20] reported only 3.4% due to advanced production practices and HACCP systems. In Spain, *Salmonella* infection levels reached 8.3%, according to Shahbazi *et al*. [21], likely due to the absence of selective enrichment methods for isolating bacteria. In Albania, the prevalence rate stands at 11.2% [22], attributed to effective control over *Salmonella* infection sources.

Our findings indicate that critical points of *Salmonella* contamination in poultry enterprises in Kazakhstan arise at various stages of production and transportation, with a vertical transmission pathway suggested by a 3.2% detection rate in incubation eggs. This finding aligns with earlier studies and suggests that *Salmonella* can be transmitted from breeding flocks to offspring, as supported by the 30% contamination rate in adult birds from cloacal samples. Secondary contamination risk is also present, particularly in equipment and work surfaces, with contamination rates reaching 4.25%. This observation is consistent with the study by Brasão *et al*. [23], who found *Salmonella* presence in 24.9% of poultry faeces, 22.7% of poultry organs and 24.3% of poultry meat and egg samples, indicating multiple potential contamination points across poultry operations.

On SS agar, *Salmonella* colonies presented as round, colourless formations with dark centres, a distinctive feature aiding in differentiation from other intestinal pathogens. On XLD agar, colonies appeared transparent with black centres due to *Salmonella*’s ability to decarboxylate lysine and reduce sulphur, forming iron sulphide precipitates. These characteristics align with findings by Obe *et al*. [24], who observed similar colony morphology on SS agar. Biochemical profiling on triple sugar iron agar further confirmed typical *Salmonella* attributes: alkaline growth with acidic reactions and H2S production, alongside positive reactions for citrate and sulphate and negative for urease and indole.

Among the various *Salmonella* serotypes identified in Kazakh poultry farms, *S*. *gallinarum-pullorum* was the most prevalent, accounting for 61.8% of isolates. This serotype presents a significant threat to poultry health due to its association with severe diseases and consequential economic losses. Effective control measures are essential to curtail the spread of this pathogen, which poses a substantial risk to poultry enterprises. Zeng *et al*. [25] found varying prevalence rates among serovars in China, identifying 14 *S*. *enteritidis* samples (11.9%) and 15 *S*. *typhimurium* samples (15.6%) through multiplex PCR among their 97 isolates, with the remaining isolates belonging to other *Salmonella* species (spp.) serovars.

Roy *et al*. [26] and Shen *et al*. [27] reported that *S. enteritidis* constitutes ~80% of cases in Turkey, a figure significantly higher than those observed in other countries. In China, *S. enteritidis* prevalence in chicken carcasses stands at 40.3% [28], 43% in Iran [29] and 67.8% in India [30], emphasizing its global adaptability and resilience. The high infectivity and adaptability of *S. enteritidis*, including its ability to colonize the gastrointestinal tract and reproductive organs of birds, necessitate focused research in Kazakhstan, as this serotype poses a considerable risk for poultry-associated salmonellosis in the region.

Antimicrobial usage regulations in Kazakhstan allow for the preventive use of antibiotics in asymptomatic animals, particularly on farms experiencing salmonellosis outbreaks. However, this unrestricted use of antimicrobials as both therapeutic and prophylactic agents, as well as growth stimulants, contributes significantly to the rise of antimicrobial resistance. Residual antibiotic contamination from unregulated disposal of pharmaceutical waste also facilitates the spread of resistance, as discussed by Chen *et al*. [31]. The prevalence of antimicrobial resistance is particularly worrisome, as it exacerbates the challenge of controlling pathogenic *Salmonella* strains. Kamal *et al*. [32] highlight that such practices result in resistant microbial populations, complicating treatment efforts in veterinary and human medicine.

Our study observed that *Salmonella* isolates exhibited resistance to a broad spectrum of antibiotics, including streptomycin, kanamycin and rifampicin, which indicates reduced effectiveness of critical antimicrobial drugs such as ansamycins. Notably, resistance was more prevalent to first-generation aminoglycosides and tetracyclines, while resistance to beta-lactams and glycopeptides was less common. These resistance patterns differ from those observed by Ohashi *et al*. [33] and Das *et al*. [34] in Egypt, where 100% of isolates were aminoglycoside-resistant, and Korzeniowski *et al*. [35] in the UK, where resistance rates reached 26.27% for tetracyclines, 23.72% for sulphonamides and 21.43% for ampicillin. Such variations emphasize the necessity of further genomic research to elucidate resistance mechanisms and identify resistance genes and integrons. Understanding these genetic components is crucial for formulating effective control strategies to mitigate the spread of resistant *Salmonella* in Kazakhstan’s poultry industry.

The biofilm formation capacity of *Salmonella* strains, particularly *S. gallinarum-pullorum*, was confirmed through experiments in this study. Our results indicate that this serotype exhibits a high tendency to adhere to surfaces and form biofilms, a key survival mechanism that aids persistence within the poultry farming environment. Biofilm formation serves as a protective barrier, enhancing bacterial resilience against environmental stressors, disinfectants and antibiotics. This observation is supported by Rakkith *et al*. [36] and Naz *et al*. [37], who demonstrated that *Salmonella* isolates from poultry meat and eggs form robust biofilms at both 30 and 37 °C. Biofilm-forming capacity significantly increases *Salmonella*’s resistance and survival rates, especially under suboptimal sanitation conditions.

Voss-Rech *et al*. [38] found that most *Salmonella* isolates, regardless of origin, exhibited strong biofilm formation at 37 °C over 48 h, with *S. typhimurium* displaying greater biofilm potential than *S. enteritidis* under optimal conditions. For Kazakhstan, this indicates a need for enhanced biosecurity protocols specifically targeting biofilm-forming strains, as traditional disinfection and sanitation practices may prove ineffective against these resilient bacterial populations.

*Salmonella* infections in poultry lead to substantial economic and health consequences. Economically, poultry farms face direct losses from infected flocks due to increased morbidity and mortality rates, which reduce the availability of market-ready birds [39]. For human health, *Salmonella* is a leading cause of foodborne illness, often transmitted through contaminated poultry products. Infections can range from mild gastrointestinal symptoms to severe, invasive cases that require hospitalization. Vulnerable groups, including young children, the elderly and immunocompromised individuals, face particularly high risks. The rise of antibiotic-resistant *Salmonella* strains has further complicated treatment, leading to prolonged illness, higher healthcare costs and an increased burden on healthcare systems [40,41]. These infections also carry societal costs, as affected individuals may lose workdays, reducing productivity and adding indirect economic strain. In regions with limited healthcare infrastructure, managing *Salmonella* outbreaks is especially challenging, placing an additional strain on public health resources. Reducing *Salmonella* infections through improved biosecurity and control measures is essential to lessen both the economic losses for poultry producers and the health risks for consumers, ultimately contributing to better food safety and public health outcomes.

The prevalence of *Salmonella* in Kazakhstan’s poultry products at 11.5% represents a moderate risk compared to global standards, falling between higher rates in China and Egypt and lower rates in Brazil. *S. gallinarum-pullorum* emerged as the dominant serotype, accounting for 61.8% of isolates and posing significant threats to poultry health and economic stability. The study revealed concerning patterns of antibiotic resistance, particularly to first-generation aminoglycosides and tetracyclines, highlighting the consequences of unrestricted antimicrobial use in Kazakhstan’s poultry industry. The research confirmed the strong biofilm-forming capacity of *Salmonella* strains, especially *S. gallinarum-pullorum*, which enhances bacterial survival and resistance to conventional sanitization methods, necessitating improved biosecurity protocols.

## Conclusions

The primary objective of this study was to systematically identify and characterize *Salmonella* strains, particularly biofilm-forming types, within industrial poultry enterprises in Kazakhstan. This objective was achieved by collecting and analysing 660 samples from multiple points in the poultry production process, including cloacal flushes, feed, shoe covers and water sources. *Salmonella* was detected in 11.5% of samples, with notable contamination in cloacal flushes, underscoring the critical role of poultry as a reservoir of this pathogen. The most common isolates were *S. gallinarum-pullorum* (61.8%), followed by *S. typhimurium* (18.4%) and *S. enteritidis* (14.5%). The high prevalence of biofilm-forming capabilities among *S. gallinarum-pullorum* isolates (48.9%) emphasizes the persistence and resilience of this pathogen in poultry production environments, which has significant implications for infection control and hygiene practices.

The study’s investigation of antimicrobial resistance revealed concerning patterns, particularly among *S. typhimurium* strains, which exhibited higher resistance levels compared to other serotypes. The identification of biofilm-forming capabilities among isolates, especially in *S. gallinarum-pullorum*, where 48.9% were strong biofilm producers, explains the persistence of these pathogens in poultry facilities despite routine sanitization efforts. The virulence assessment demonstrated that isolates from clinical cases required lower infective doses (103–105 c.f.u.) compared to environmental isolates (107–109 c.f.u.), indicating adaptation mechanisms that enhance pathogenicity within host organisms.

The findings of this study carry significant implications for public health, food safety and the poultry industry, particularly regarding biofilm-forming *Salmonella* strains that enhance bacterial survival on production surfaces and increase cross-contamination risks, making disinfection more challenging. The higher antimicrobial resistance observed in *S. typhimurium* strains compared to * S. gallinarum-pullorum* emphasizes the necessity for regular antibiotic sensitivity monitoring among *Salmonella* serovars to inform treatment protocols, while effective sanitation practices and judicious antibiotic use are crucial for controlling resistant strains. The implementation of systematic HACCP approaches to identify contamination hotspots can strengthen biosecurity measures and minimize *Salmonella* spread within poultry farms. Given the study’s limitations in geographic scope and seasonal coverage, future research should expand to include more regions and assess seasonal variations in *Salmonella* prevalence, while investigating genetic mechanisms of resistance and alternative control measures such as probiotics and vaccines, and biofilm prevention methods will be essential for developing comprehensive strategies to reduce *Salmonella* contamination and protect public health.

## Abbreviations

HACCP: Hazard Analysis and Critical Control Points
MPA: meat-peptone agar
SS: *Salmonella*-Shigella
TSB: tryptic soy broth
XLD: xylose-lysine-deoxycholate
